# Divergent Effects of Dioxin- or Non-Dioxin-Like Polychlorinated Biphenyls on the Apoptosis of Primary Cell Culture from the Mouse Pituitary Gland

**DOI:** 10.1371/journal.pone.0146729

**Published:** 2016-01-11

**Authors:** Francesco Raggi, Dania Russo, Claudio Urbani, Chiara Sardella, Luca Manetti, Daniele Cappellani, Isabella Lupi, Luca Tomisti, Enio Martino, Claudio Marcocci, Fausto Bogazzi

**Affiliations:** Department of Clinical and Experimental Medicine, Section of Endocrinology, University of Pisa, Pisa, Italy; The University of Iowa, UNITED STATES

## Abstract

Polychlorinated biphenyls (PCBs) can disrupt the endocrine function, promote neoplasms and regulate apoptosis in some tissues; however, it is unknown whether PCBs can affect the apoptosis of pituitary cells. The study evaluated the effect of PCBs on the apoptosis of normal pituitary cells and the underlying mechanisms. Primary cell cultures obtained from mouse pituitary glands were exposed to Aroclor 1254 or selected dioxin-like (PCB 77, PCB 126) or non-dioxin-like (PCB 153, PCB 180) congeners. Apoptosis was evaluated by Annexin V staining, DNA fragmentation, and TUNEL assay. Both the expression and activity of caspases were analyzed. Selective thyroid hormone receptor (TR) or aryl-hydrocarbon receptor (AhR) or CYP1A1 antagonist were used to explore the mechanisms underlying PCBs action. Our results showed that Aroclor 1254 induced the apoptosis of pituitary cells as well as the final caspase-3 level and activity through the extrinsic pathway, as shown by the increased caspase-8 level and activity. On the other hand, the intrinsic pathway evaluated by measuring caspase-9 expression was silent. The selected non-dioxin-like congeners either increased (PCB 180) or reduced (PCB 153) pituitary cell apoptosis, affecting the extrinsic pathway (PCB 180), or both the extrinsic and intrinsic pathways (PCB 153), respectively. In contrast, the dioxin-like congeners (PCB 77 and PCB 126) did not affect apoptosis. The anti-apoptotic phenotype of PCB 153 was counteracted by a TR or a CYP1A1 antagonist, whereas the pro-apoptotic effect of PCB 180 was counteracted by an AhR antagonist. The induced apoptosis of Aroclor 1254 or PCB 180 was associated with a reduction of cell proliferation, whereas the decreased apoptosis due to PCB 153 increased cell proliferation by 30%. In conclusion, our data suggest that non-dioxin-like PCBs may modulate apoptosis and the proliferation rate of pituitary cells that have either pro- or anti-apoptotic effects depending on the specific congeners. However, the impact of PCBs on the process of pituitary tumorigenesis remains to be elucidated.

## Introduction

Polychlorinated biphenyls (PCBs) are persistent pollutants, which can disrupt the endocrine function [1], and promote the incidence of tumors [2, 3].

There is increasing evidence that the hypothalamic-pituitary axis may be targeted by chemicals with endocrine disruption activities [4]. Some endocrine disruptors interact with native hormone receptors, acting as either antagonists or weak agonists [5–7]. Specifically, dioxin and some PCBs with a dioxin-like structure may bind to the aryl-hydrocarbon receptor (AhR) [8]. On the other hand, some PCBs with a non-dioxin-like structure can activate or suppress the gene expression regulated by the thyroid hormone, interacting with the thyroid hormone receptor (TR) [9].

In addition to the disruption of the endocrine function through the direct interaction with hormone receptors, PCBs can affect the endocrine system by modulating apoptosis [10]. However, little information is available concerning the influence of PCBs on apoptosis in the endocrine system, and specifically in the pituitary. It has been reported that in testes the non-dioxin-like PCB 132 may reduce apoptosis at low concentrations, and increase apoptosis at high doses [11].

The regulation of apoptosis is a key step in the early phase of tumorigenesis [12], since it promotes the progression of predisposed cells [13]. Pollutants, including PCBs, have been associated with the induction of neoplasms through AhR and cytochrome P450-1A1 (CYP1A1) regulation [14]. PCBs both enhance or reduce apoptosis, depending on the cell system and PCB congener [15–18]. Overall, the published data converge on the anti-apoptotic effect of the PCB 153 congener in various cell systems [19–22]. However, data regarding the effects of PCBs on the apoptosis of the pituitary gland are lacking.

Pituitary adenomas are usually benign intracranial tumors representing about 20% of intracranial neoplasms [23]. On the clinical grounds, pituitary adenomas may lead to syndromes related to the hypersecretion of the pituitary hormone to the local mass effect of the lesion (e.g. headaches, visual defects), and/or hypopituitarism [23]. Prolactinomas are the most frequent subtype of pituitary adenomas, followed by non-functioning, GH-secreting, and ACTH-secreting adenomas [23].

The pathogenesis of these tumors is complex and still largely unknown, although a pathophysiological role of hereditary predisposition, somatic mutations and endocrine factors has been proposed [24].

Few data are available regarding the impact of environmental contaminants and pollution on the etiology of pituitary adenomas. However, a recent epidemiological study performed in the South of Italy showed that the prevalence of GH-secreting tumors was higher in a highly polluted area respect to the prevalence observed in nearby areas [25].

The aim of the present study was to evaluate whether a mixture of PCBs (Aroclor 1254) or individual congeners with a dioxin- or non-dioxin-like structure, affect the apoptosis of a primary cell culture obtained from the mouse pituitary gland.

## Materials and Methods

### Study design

The study was designed to investigate the effect of PCBs on the spontaneous apoptosis of normal pituitary cells. Pituitary cell cultures were obtained from the pituitary of 8–12 week old C57BL/6J x CBA male mice. To perform the cell signaling experiments on primary pituitary gland cells, only male mice were utilized as donors to avoid gender related effects [26]. In addition, we examined whether the changes in apoptosis were associated with reactive oxygen species (ROS) production or involved TR, AhR or CYP1A1, the main targets of PCBs. Lastly, we evaluated whether changes in apoptosis were associated with changes in cell proliferation. Preliminary experiments were carried out to assess the toxic dose of the PCBs used, by the 3-(4,5-dimethylthiazol-2-yl)-2,5-diphenyltetrazolium bromide (MTT) assay, according to a previous study [27]. In brief, pituitary cell cultures were treated for 24 or 48 hours with Aroclor 1254 or individual congeners (PCB 77, PCB 126, PCB 153, PCB 180) in the concentration range of 1–50 μM (data not shown). The conditions selected for the apoptotic evaluation were 24h exposure time and 10 μM PCB concentration, which were not associated with a significant reduction in cell viability (<3%).

Several experiments were carried out to investigate the early and late events of cellular apoptosis. The exposure to phosphatidylserine by the Annexin V staining assay or the DNA fragmentation level were analysed by both ELISA and TUNEL tests. In addition, analyses the expression and activity of proteins involved in the apoptosis pathway were measured. For Annexin V staining assay and protein expression evaluation, cells were seeded into a 35mm cell culture dish coated with poly-l-lysine (Sigma-Aldrich, St. Louis, MO, USA) at a density of 5x10^5^ cells in a 1 ml complete culture medium. This consisted of Neurobasal-A supplemented with B-27 supplement (1:50) (Life Technologies, Carlsbad, CA, USA), 0.5mM L-glutamine, 10% heat-inactivated fetal bovine serum, 2% Penicillin-Streptomycin and 1% Amphotericin B. For DNA fragmentation, 5-Bromo-2’-deoxyuridine (BrdU) proliferation, caspases activity and ROS production evaluations, cells were seeded and cultured in adhesion into 96-well tissue culture plates coated with poly-l-lysine (Sigma-Aldrich, St. Louis, MO, USA) at a density of 5x10^3^ cells/well in a 100 μl complete culture medium. For the immunofluorescence or TUNEL tests, cells were distributed into 8-chamber cell culture slides (BD Biosciences, San Jose, CA, USA) coated with poly-l-lysine (Sigma-Aldrich, St. Louis, MO, USA), at a density of 2x10^5^ cells/slide in a 250 μl complete culture medium. In all the experiments, after a 3-day culture period [28], cells were incubated for 24 hours with fresh serum-free medium containing the test substances at the appropriate concentrations or the corresponding control vehicle.

### Primary cell culture of mouse pituitary

Primary cultures of pituitary cells were obtained from normal mice, following standard procedures [29] with minor changes. C57BL/6J x CBA male mice (8–12 weeks old) were euthanized by CO_2_ and the individual pituitaries were dissected and stored on ice in Hank’s balanced salt solution (Sigma-Aldrich, St. Louis, MO, USA). Pituitaries were dispersed to individual cells by enzymatic treatment for 1h at 37°C with 0.25% Trypsin type II-S (Sigma-Aldrich, St. Louis, MO, USA) in Neurobasal-A medium (Life Technologies, Carlsbad, CA, USA) with 2% penicillin-streptomycin (Sigma-Aldrich, St. Louis, MO, USA), in a humidified atmosphere of 95% air and 5% CO_2_.

**Fig 1:**
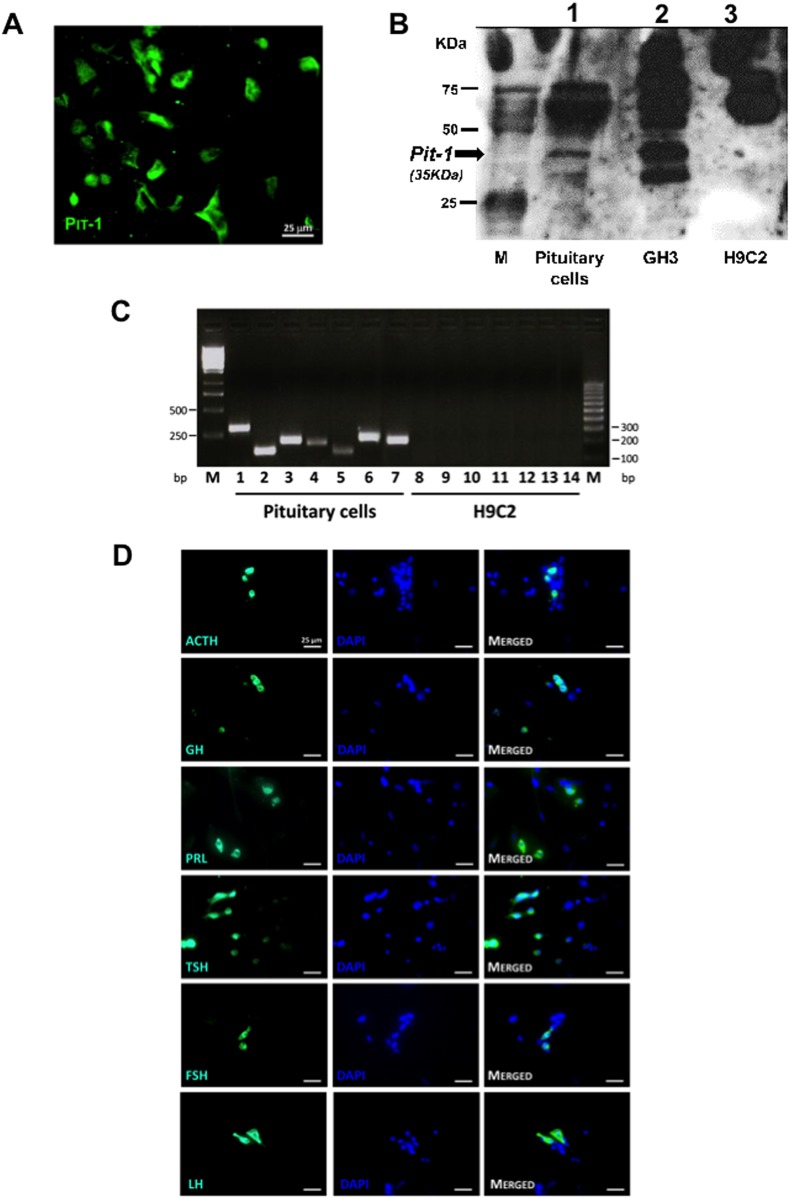
Pituitary primary cells culture. Primary cells were obtained from mouse pituitaries as detailed in the Materials and Methods. The pituitary cells was identified before each experiment by immunofluorescence (A) and Western blot (B) for Pit-1. Each cell lineage was confirmed by RT-PCR (C) and immunofluorescence (D) for each pituitary hormone. (A, D) Immunofluorescence detection of Pit-1 and pituitary hormones, respectively, in primary cells. 4,6-diamidino-2-phenylindole (DAPI) (blue stain) was used as a nuclear marker. Merged pictures showed cells simultaneously stained with pituitary hormones and DAPI. Magnification, 40X; bars = 25μm (B) Representative blot of Pit-1 protein (filled arrow) (line1); GH3 cells were used as a positive control expressing Pit-1(line 2); a non-pituitary cell line (H9C2) was used as a negative control (line 3) (C) Representative 2% agarose gel of PCR-amplified products. Abbreviations: M, marker; 1, Pit-1 330bp; 2, POMC 149bp; 3, FSHβ 218bp; 4, LHβ 196bp; 5, GH 149bp; 6, PRL 244bp; 7, TSHβ 224bp; 8–14, negative control amplification lines.

The viable cells (> 97%), evaluated by Trypan blue dye exclusion assay, were plated with complete culture medium in a humidified atmosphere of 95% air and 5% CO_2_. The pituitary cells were identified by hematoxylin-eosin inclusion (data not shown) followed by the evaluation of the selective expression of Pit-1 through immunofluorescence and Western blot (Fig 1A and 1B). In addition, to verify that all pituitary cell lineages were represented, the expression of each pituitary hormone was evaluated both by reverse(RT)-PCR and by immunofluorescence (Fig 1C and 1D). GH3 cell line, derived from GH-secreting pituitary adenoma, was used as a positive control for Pit-1 expression, and H9C2 cell line (rat myoblast) was used as a negative control.

All procedures on C57BL/6J x CBA male mice were conducted in accordance with the National Institute of Health guidelines for the use of experimental animals [30]. The study protocol was approved by the local Board for Animals Experimentation at the University of Pisa.

### Reverse (RT)-PCR

Total RNA was extracted from primary pituitary cells using the standard TRIzol (Invitrogen, Carlsbad, CA, USA) method according to the manufacturer’s protocol. Extracted RNA quality was assessed using the RNA 6000 Nano LabChip assay and an Agilent 2100 Bioanalyzer instrument (Agilent Technologies, Santa Clara, CA, USA). Total RNA (1 μg) was transcribed into cDNA using Superscript III reverse transcriptase (Invitrogen, Carlsbad, CA, USA) and pd(N)6 random hexamer (GE Healthcare, Milan, Italy) in a final volume of 20 μl, according to the manufacturer’s protocol, as reported [31]. The primers used to verify the presence of Pit-1, FSHβ, LHβ, POMC, TSHβ, PRL, and GH in cultured pituitary cells were designed using mouse mRNA sequences, as previously described [29, 32], and are listed in Table 1. The thermocycling conditions of PCR were: 95°C for 30s (denaturation), 60°C or 58°C depending on primer requirements for 30s (annealing), and 72°C for 1 min (extension).

**Table 1:** Primers sequences used in RT-PCR analysis.

Gene	Primer Sequence (5’to 3’)	Product Size (bp)	Annealing Temp (°C)	Cross Intron
GH	F: TACCTGCGGGTCATGAAG	149	60	yes
	R: GATGCATCTTAATTTTATTAGGAC			
PRL	F: TGTTCAGCCTCTGCCAATCTGTTC	244	60	yes
	R: AAGAACTTCCGGAGGGACTTTCAG			
FSH	F: GAAGGAAGAGTGCCGTTTCTGCAT	218	60	no
	R: TGCCACAGTGACATTCAGTGGCTA			
LH	F: TGTCCTAGCATGGTCCGAGT	196	60	yes
	R: GACCCCCACAGTCAGAGCTA			
TSH	F: GGCAAACTGTTTCTTCCCAA	224	58	no
	R: TAGAAAGACTGCGGCTTGGT			
POMC	F: TAGATGTGTGGAGCTGGTGC	149	60	yes
	R: CAGTCAGGGGCTGTTCATCT			
Pit-1	F: AGTTTAAGCAGGAACTCAGGCGGA	330	60	yes
	R: TCCTCTTCCTTTCGTTTGCTCCCA			

All PCR-amplified products were visualized under ultraviolet light on a 2% agarose gel containing ethidium bromide. 1 kilobase (Kb) and 100 base pairs (bp) DNA Ladder (M-Medical, Milan, Italy) were used to determine the product size. All PCR fragments were sequenced to confirm identity.

### Immunofluorescence

Immunofluorescence was carried out as previously described [33]. In brief, cells were distributed and cultured on 8-chamber cell culture slides (BD Biosciences, San Jose, CA, USA), as described in the study design section. The cells were then exposed overnight at 4°C to 1:200 pituitary hormones or Pit-1 primary antibodies and stained with Alexa Fluor 488-conjugated streptavidin secondary antibody (Life Technologies, Carlsbad, CA, USA). 4,6-diamidino-2-phenylindole (DAPI) (Life Technologies, Carlsbad, CA, USA) was used as a nuclear marker.

### Chemicals

Aroclor 1254 and individual congeners (PCB 77, PCB 126, PCB 153, PCB 180) were purchased from VWR International (Radnor, PA, USA). The purity of each PCB congener was higher than 99%, as assayed by gas-chromatography. Thyroid hormone receptor antagonist (1–850), α-Naphthoflavone (α-NF) (AhR inhibitor) and 7-Hydroxyflavone (HF) (CYP1A1 inhibitor) were purchased from Santa Cruz Biotechnology (Dallas, TX, USA). cis-diammineplatinum(II)dichloride (Cisplatin), Tumor Necrosis Factor-α (TNF-α), 3,3’,5 triiodothyronine (T3), 2,3,7,8-Tetrachlorodibenzo-p-dioxin (TCDD), Cycloheximide (CHX), Colchicine, Hydrogen Peroxide (H_2_O_2_) and Ascorbic Acid (vitamin C) were purchased from Sigma-Aldrich (St. Louis, MO, USA). Ac-DEVD-CHO (caspase-3 inhibitor) and Ac-IETD-CHO (caspase-8 inhibitor) were purchased from BD Biosciences (San Jose, CA, USA). Ac-LEHD-CHO caspase-9 inhibitor was purchased from Alexis Biochemicals (San Diego, CA, USA). Chemicals were dissolved in dimethyl sulfoxide (DMSO), which was used as a vehicle (0.1% final concentration), in the cellular apoptosis experiments.

### Apoptosis

Apoptosis was evaluated by methods assessing either early or late events. Specifically, phosphatidylserine exposition in the outer leaflet of the plasma membrane was determined in the initial apoptotic phase, using the Annexin V-FITC Apoptosis Detection kit (Sigma-Aldrich, St. Louis, MO, USA) [34]; DNA fragmentation was determined in the final stage of apoptosis, by both the cell death detection ELISA plus kit (Roche Applied Science, Penzberg, Germany) and the terminal deoxynucleotidyltransferase mediated dUTP nick end labeling (TUNEL) assay (Roche Applied Science, Penzberg, Germany) [34, 35]. For the Annexin V-FITC assay, pituitary cells, treated as described in the study design section, were collected and resuspended in 500 μl binding buffer containing 5 μl fluorescein isothiocyanate (FITC) conjugated annexin V and 10 μl propidium iodide solutions, according to manufacturer’s instructions. After incubation in the dark at room temperature for 10 min, the suspensions were processed in FACS Calibur flow cytometer (BD Biosciences, San Jose, CA, USA). Ten thousand events were recorded for each treatment group and analysed using the CellQuest software (BD Biosciences, San Jose, CA, USA).

For the DNA fragmentation assay, cells from mouse pituitary were cultured and treated as described in the study design section. To account for the percentage of dead cells due to sample handling and processing, cell viability was evaluated in 96-well plates after 24, 48 and 72 h from seeding, using the Trypan blue exclusion assay and the Differential Nuclear Staining (DNS) assay, as previously reported [27, 36], with minor changes. Cell viability was ≥97% (data not shown). After the PCBs treatments, supernatant culture media were removed and a lysis buffer was added. The cellular lysates were centrifuged for 10 min to separate the cytoplasmic fraction from intact nuclei. The supernatant was then transferred to a 96-well streptavidin-coated plate, with an anti–histone-biotin and anti–DNA peroxidase mixture. The plate was incubated for 2 hours, washed, and 2,2′-azinobis[3-ethylbenzothiazoline-6-sulfonic acid]-diammonium salt (ABTS) was added for 15 minutes. Colorimetric determination was performed at 405 nm using a microplate reader.

For the TUNEL assay, primary cells cultures were fixed with 4% paraformaldehyde in phosphate buffer saline (PBS), pH 7.4, for 20 min at room temperature, washed with PBS for 30 min and incubated with permeabilization solution (0.1% Triton X-100 in 0.1% sodium citrate) for 2 min on ice. Cells were washed twice and incubated in a humidified atmosphere with TUNEL reaction mixture for 60 min at 37°C in the dark and finally analyzed under a fluorescence microscope with a 488 nm excitation wavelength.

Apoptosis was quantified by staining the cells in four fields randomly chosen from each well of 8-chamber slides with TUNEL (green stain) and with the nuclear marker, 4,6-diamidino-2-phenylindole (DAPI) (blue stain). Cisplatin (32 μM) was used as a positive control, according to previous studies [27, 37].

### Cell extracts

Primary cell cultures treated as described in the study design section, were washed twice with ice-cold PBS, collected by gentle trypsinization, pelleted, and homogenized with a lysis buffer containing 150 mM NaCl, 10 mM Tris-HCl (pH 7.4), 1 mM EGTA, 1 mM EDTA, 1% Triton X-100, and protease inhibitors (1 μg/ml aprotinin, 1 μg/ml pepstatin A, 1 μg/ml leupeptin and 1 mM PMSF). After incubation on ice for 30 min and subsequent centrifugation, supernatants were recovered and stored at -80°C. Protein concentrations in the supernatant fractions were measured by a commercially available kit, based on the Bradford method (Bio-Rad Laboratories, Hercules, CA, USA).

### Antibodies

The following antibodies were used: caspase-9 (H170) rabbit polyclonal IgG antibody, caspase-3 (E-8) rabbit polyclonal IgG antibody, caspase-8 p20 (H-134) rabbit IgG antibody, cytochrome c (7H8) mouse monoclonal IgG antibody, TRADD (H-278) rabbit polyclonal IgG antibody, β-actin (C2) mouse monoclonal IgG antibody, Pit-1(X-7) rabbit IgG antibody, ACTH (B427) mouse monoclonal IgG antibody, TSHβ (M-16) goat polyclonal IgG antibody, Prolactin (C-17) goat polyclonal IgG antibody, FSHβ (C-19) goat polyclonal IgG antibody, LHβ (R-16) goat polyclonal IgG antibody, all from Santa Cruz Biotechnology (Dallas, TX, USA). Anti goat, rabbit or mouse IgG Ab-horseradish peroxidase (HRP)-conjugated (Santa Cruz Biotechnology (Dallas, TX, USA) and Alexa Fluor 488-conjugated streptavidin (Life Technologies, Carlsbad, CA, USA) were used as secondary antibodies.

### ROS production

Quantitative ROS generation was measured using the oxidant-sensitive probe dichlorofluorescein (H2DCF-DA) assay, as previously reported [38]. Pituitary primary cells were treated as described in the study design. At the end of the incubation period, the medium was replaced by 100 μl/well HBSS containing 10 μM 2’,7’–dichlorofluorescin diacetate (DCFDA) (Invitrogen, Carlsbad, CA, USA) and the cells were incubated for 30 min a 37°C in the dark, according to the manufacture’s protocol. After diffusion into the cell, DCFDA was deacetylated by cellular esterases to a non-fluorescent compound, which was later oxidized by ROS into highly fluorescent 2’,7’–dichlorofluorescein (DCF). The fluorescence intensity, which was proportional to ROS levels, was measured at 527 nm (excitation wavelength 495 nm), using a microplate reader (FLUOstar Omega, BMG Labtech, Ortenberg, Germany). 10 μM Hydrogen Peroxide (H_2_O_2_) was used as a positive control to induce oxidative stress in the cell cultures, whereas 1 mM Ascorbic Acid (vitamin C) was used as antioxidant, as previously described [39, 40].

### Western blotting

Total (25 μg) cell extracts were resolved on a 12% SDS-PAGE, transferred onto nitrocellulose membrane, and stained with Ponceau red to verify the amount of proteins per lane. The transferred proteins were incubated overnight at 4°C in 50% Tris-buffered saline (200 mM Tris-HCl (pH 7.6) and 1.4 mM NaCl) as well as 50% TTBS (Tris-buffered saline with 0.05%, Tween 20) containing 5% non-fat dry milk. They were then incubated with the appropriate primary antibody (1:200) overnight at 4°C. After TTBS washing, an IgG HRP-conjugated secondary antibody (1:1000) was added for 1h at RT. Linked proteins were detected using an enhanced chemiluminescence detection system (Bio-Rad Laboratories, Hercules, CA, USA). Membranes were incubated at 70°C for 10 min in a 5 mM Tris-HCl stripping buffer (pH 6.8), 2% SDS, 0.8% β-mercaptoethanol and reprobed for β-actin for loading normalization. Films were scanned with a densitometer (Bio-Rad Laboratories, Hercules, CA, USA) and band intensity was evaluated using Interfocus software (InterfocusGmbH, Sonnenblumenring, Mering, Germany). Each density value was normalized for loading errors (dividing by intensity of β-actin).

### Caspase activity

Caspase activity was assessed by fluorimetric or luminometric assays using the NucView 488 Caspase-3 kit for live cells (Biotium, Hayward, CA, USA) and Caspase -8 or -9 Glo kit (Promega, Milan, Italy) respectively, as previously reported [41, 42]. In brief, cells were seeded in clear bottom 96-well plates coated with poly-l-lysine (Sigma-Aldrich, St. Louis, MO, USA) at a density of 5x10^3^ cells/well and were treated as described in the study design section. Cisplatin (32 μM), a well-known pro-apoptotic inductor via caspases activation [37, 43–45] was included as a positive control. In order to confirm the signal specificity, cells were exposed to PCBs in the absence or presence of 20 μM caspase inhibitor. Specifically, Ac-DEVD-CHO, Ac-IETD-CHO, and Ac-LEHD-CHO were used as inhibitors of caspase -3, -8, and -9, respectively, as previously reported [46]. At the end of the treatment period, cells were incubated with appropriate fluorogenic (for caspase-3) or luminogenic (for caspase-8 and -9) substrate for 30 minutes at room temperature, according to the manufacturer’s instructions. Fluorescent or luminescent measurements were performed using the FLUOstar Omega microplate reader (BMG Labtech, Ortenberg, Germany).

### 5-Bromo-2’-deoxyuridine (BrdU) incorporation assay

Cell proliferation was evaluated by BrdU incorporation ELISA colorimetric assay (Roche Applied Science, Penzberg, Germany). In brief, cells were seeded in 96-well plates coated with poly-l-lysine (Sigma-Aldrich, St. Louis, MO, USA) at 5x10^3^/well and treated as described in the study design section. As a positive control, cells were treated with culture medium supplemented with 10% fetal bovine serum (FBS) [47]. Colchicine (1 μM) was used as a negative control, as previously reported [27]. At the end of the treatment period, cells were incubated with 10 μM bromodeoxyuridine (BrdU)-labelling solution per well for 4 h, dried, fixed and detected using an anti-BrdU antibody according to the manufacturer’s instructions. Finally, the absorbance was measured using a microplate reader at 450 nm with a reference wavelength of 690 nm.

### Statistical analysis

Results were expressed as mean ± SD of five independent experiments. ANOVA followed by Dunnett’s post hoc test were used to assess significant differences between PCBs treatment and control vehicle. The comparison between PCBs with and without selective inhibitor compounds was performed by the Student’s t-test: the p value was adjusted for multiple comparisons following the Bonferroni method. A p value <0.05 was considered statistically significant. All analyses were performed by Prism version 5.0 (GraphPad Software, San Diego, CA, USA).

## Results

### The effect of PCBs on the apoptosis of pituitary cells

**Fig 2:**
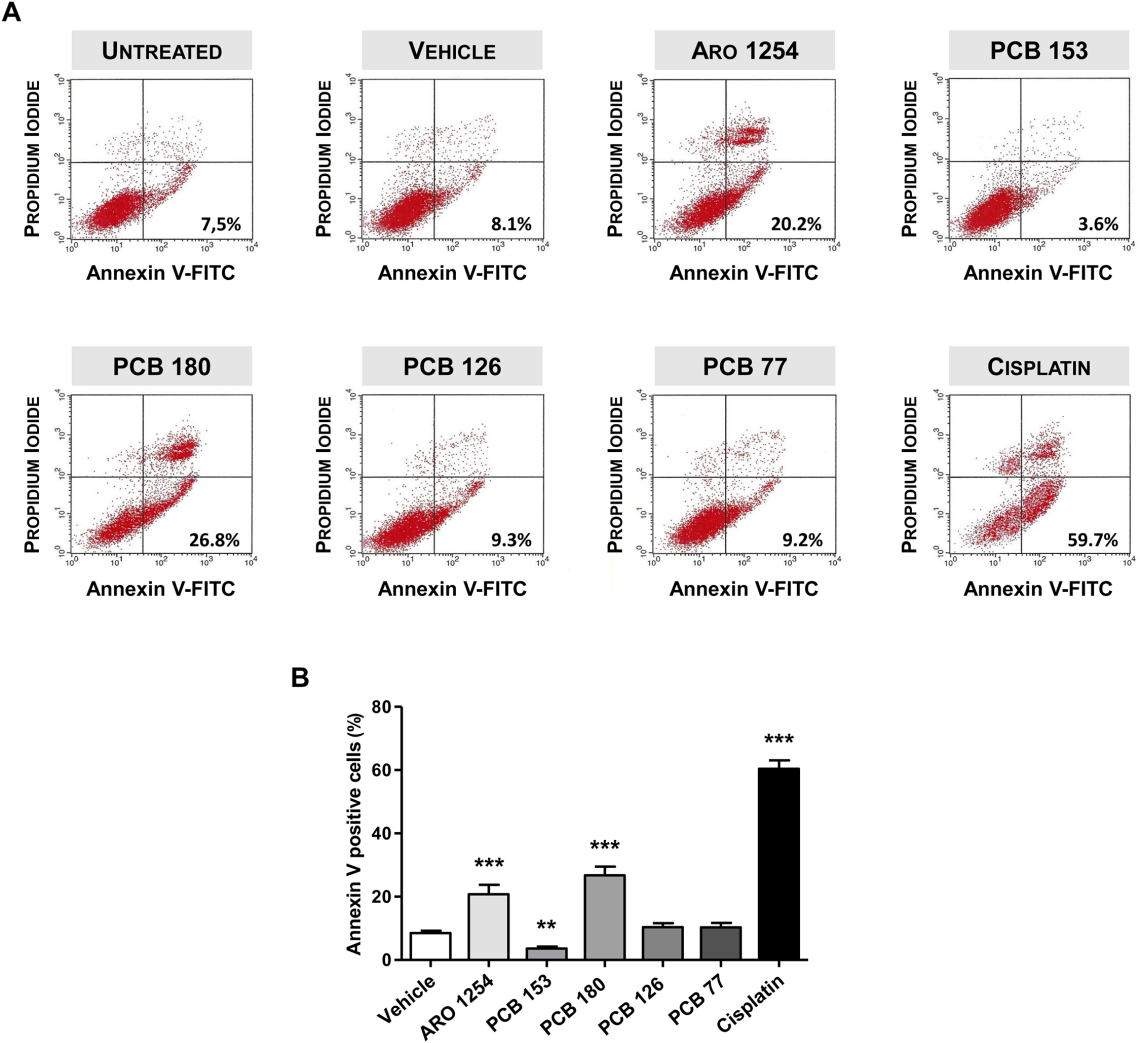
Early cellular events in apoptosis of pituitary cells during exposure to PCBs. Primary pituitary cells were incubated for 24 hours with fresh serum-free medium containing 10 μM PCBs or vehicle (DMSO 0.1%). Apoptosis was evaluated by flow cytometric analysis after co-staining of the pituitary cells with annexin V-FITC and propidium iodide (PI), as detailed in the Materials and Methods. (A) Representative flow cytometric scatter plots of Annexin V staining assay: the right bottom quadrant of each plot contains the early apoptotic cells (annexin V-FITC positive/PI negative). The PCB mixture Aroclor 1254 (ARO) induced an early phase of pituitary cell apoptosis. Two non-dioxin-like congeners reduced (PCB 153) or increased (PCB 180) apoptosis, respectively. In contrast, two dioxin-like congeners (PCB 126 and PCB 77) did not significantly affect apoptosis. Cisplatin (32 μM) was used as a positive control. The scatter plot for untreated cells is also reported. (B) Percentages of annexin V-FITC positive cells in the different treatments. Results represent the mean value ± SD of five independent experiments. **p<0.001, ***p<0.0001 compared with the vehicle, according to ANOVA followed by Dunnett’s post hoc test.

**Fig 3:**
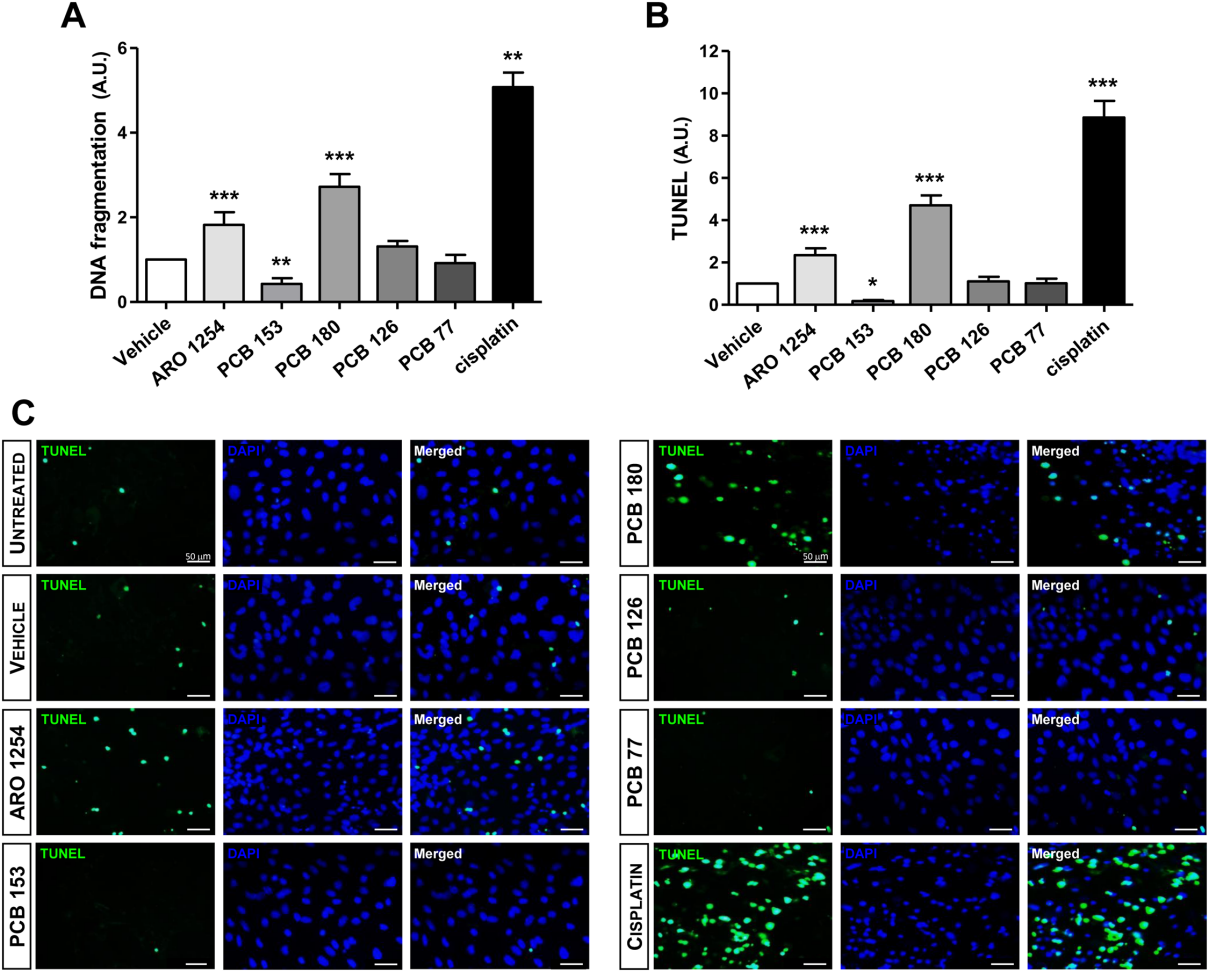
Changes in late phase of pituitary cells apoptosis during exposure to PCBs. The evaluation was performed by measuring the cellular DNA fragmentation level using the ELISA colorimetric test (A) or terminal deoxynucleotidyl transferase mediated dUTP nick end labeling (TUNEL) assay (B, C), as in the Materials and Methods. Cisplatin (32 μM) was used as a positive control. A late phase of pituitary cells apoptosis was raised by Aroclor 1254 or PCB 180, was almost suppressed by PCB 153 or left unaltered by the dioxin-like congeners PCB 126 and PCB 77. (A) Primary pituitary cells were incubated for 24 hours with fresh serum-free medium containing 10 μM PCBs or vehicle (DMSO 0.1%). Data represent the mean values ± SD of five independent experiments, each performed in quadruplicate and are expressed as arbitrary units (A.U.), assuming that the DNA fragmentation level obtained from the cells exposed to vehicle was considered as 1. **, p<0.001, ***, p<0.0001, compared with vehicle, according to ANOVA followed by Dunnett’s post hoc test. (B, C) Primary pituitary cells were treated as described in the study design. Specificity of TUNEL (green) nuclear labelling was confirmed by DAPI (blue), which selectively identifies nuclei. Merged pictures represent cells stained with both TUNEL and DAPI. Results represent the mean ± SD of five independent experiments and are expressed as arbitrary units (A.U), considering the TUNEL level obtained from the cells exposed to vehicle as 1. *, p<0.01, ***, p<0.0001 compared with vehicle, according to ANOVA followed by Dunnett’s post hoc test. Magnification, 40X; bars = 50μm.

Primary pituitary cells were exposed to Aroclor 1254 or individual congeners, with either a dioxin-like (PCB 77, PCB 126) or non-dioxin-like (PCB 153, PCB 180) structure. Early cellular events related to PCB-induced apoptosis were revealed by Annexin V assay. As shown in Fig 2A and 2B, the percentage of cells that were positive for Annexin V staining and negative for propidium iodide staining (lower right quadrant), significantly increased after treatment with Aroclor 1254 and non-dioxin-like congener PCB 180 (p<0.0001). Conversely, the other non-dioxin-like congener PCB 153 caused a significant reduction (p<0.01) in the percentage of annexin-positive cells, compared to that observed in cells not exposed to PCBs. Interestingly, no significant changes in the percentage of cells positive for annexin V were detected when the dioxin-like congeners PCB 77 or PCB 126 were used. The level of apoptosis in the late phase, evaluated by both the DNA fragmentation assay and the TUNEL assay, increased after exposure to Aroclor 1254 and to non-dioxin-like PCB 180 (Fig 3A–3C). In contrast, the treatment with PCB 153 reduced cellular apoptosis more than 50%, compared to vehicle treatment, whereas the two dioxin-like congeners PCB 77 and PCB 126 were silent (Fig 3A–3C). Cisplatin (32 μM), a known apoptosis inducer in normal [37] and cancer cells [27], was used as a positive control and caused a highly significant induction of both early and late apoptosis of pituitary cells, as expected (Fig 3A–3C). In summary, the non-dioxin-like congeners seem to be responsible for the changes in apoptosis of the pituitary cells exposed to PCBs.

### The effects of PCBs on the expression and activity of caspases

**Fig 4:**
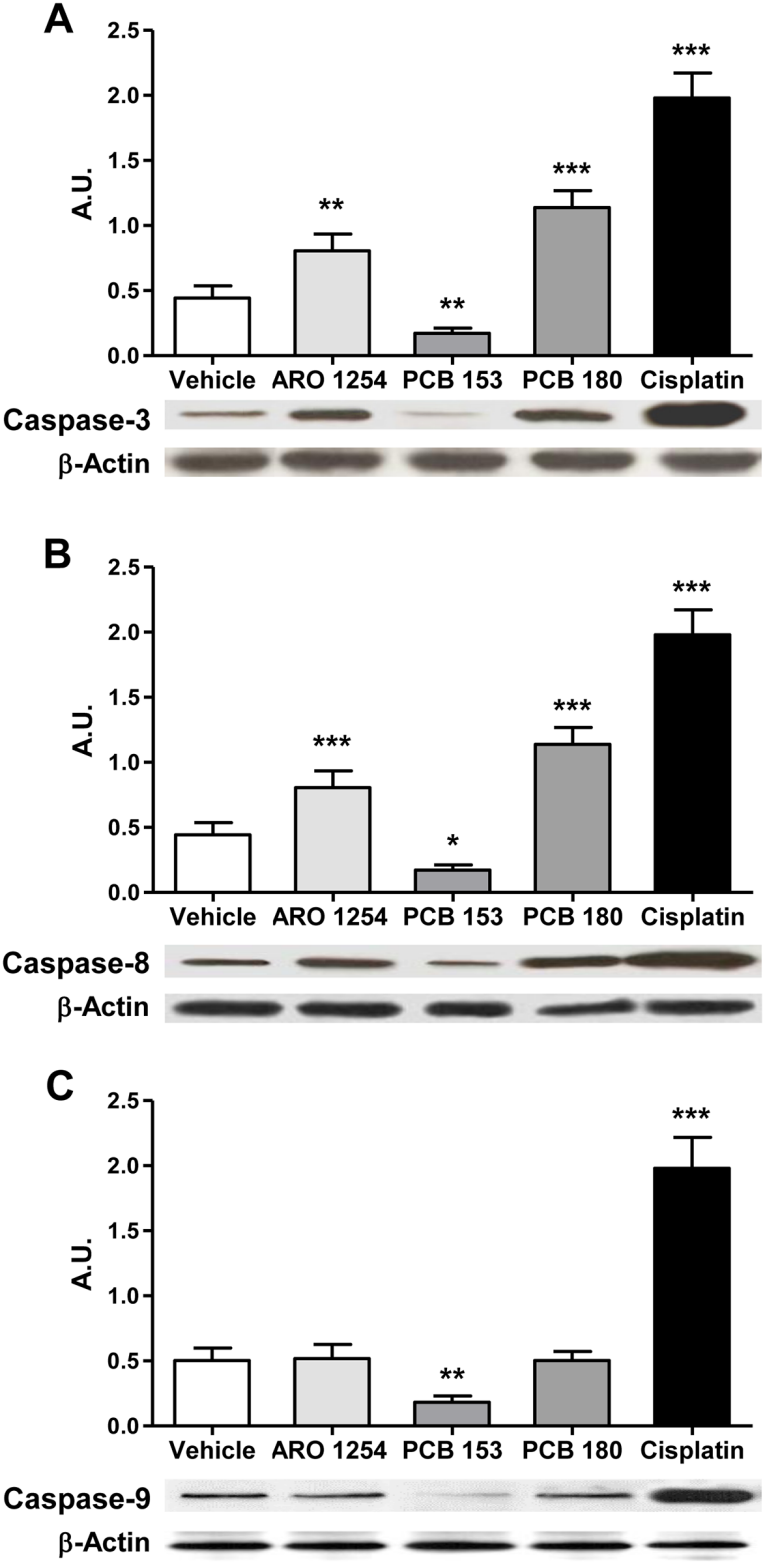
Changes in the expression of caspases during exposure to PCBs. Representative Western blot from pituitary primary cell extracts obtained as described in the Materials and Methods. Primary pituitary cells were incubated for 24 hours with fresh serum-free medium containing the test substances at a 10 μM concentration or the vehicle (DMSO 0.1%). Cisplatin (32 μM) was used as a positive control. The expression of the final effector of apoptosis, i.e. caspase-3, changed in relation to the change in apoptosis during the exposure to a mixture of PCBs or individual congeners (A). In addition, the intrinsic (i.e., mithochondrial) apoptotic pathway was not influenced by either the mixture (Aroclor 1254) or the congener PCB 180, as shown by the unchanged expression of caspase-9 (C). In contrast, the non-dioxin-like PCB 180 and Aroclor 1254 increased the expression of caspase-8 (B), suggesting the involvement of the extrinsic pathway. Interestingly, the non-dioxin-like PCB 153 reduced the expression of both caspase-8 and 9 (B, C). Data are expressed as arbitrary units (A.U.), which represent the ratio between the intensity of the band of interest and the intensity of the band corresponding to the control protein (β-actin). The mean ± SD of measurements obtained in five independent experiments, are reported. *, p<0.01, **, p<0.001, ***, p<0.0001, compared with the vehicle, according to ANOVA followed by Dunnett’s post hoc test.

**Fig 5:**
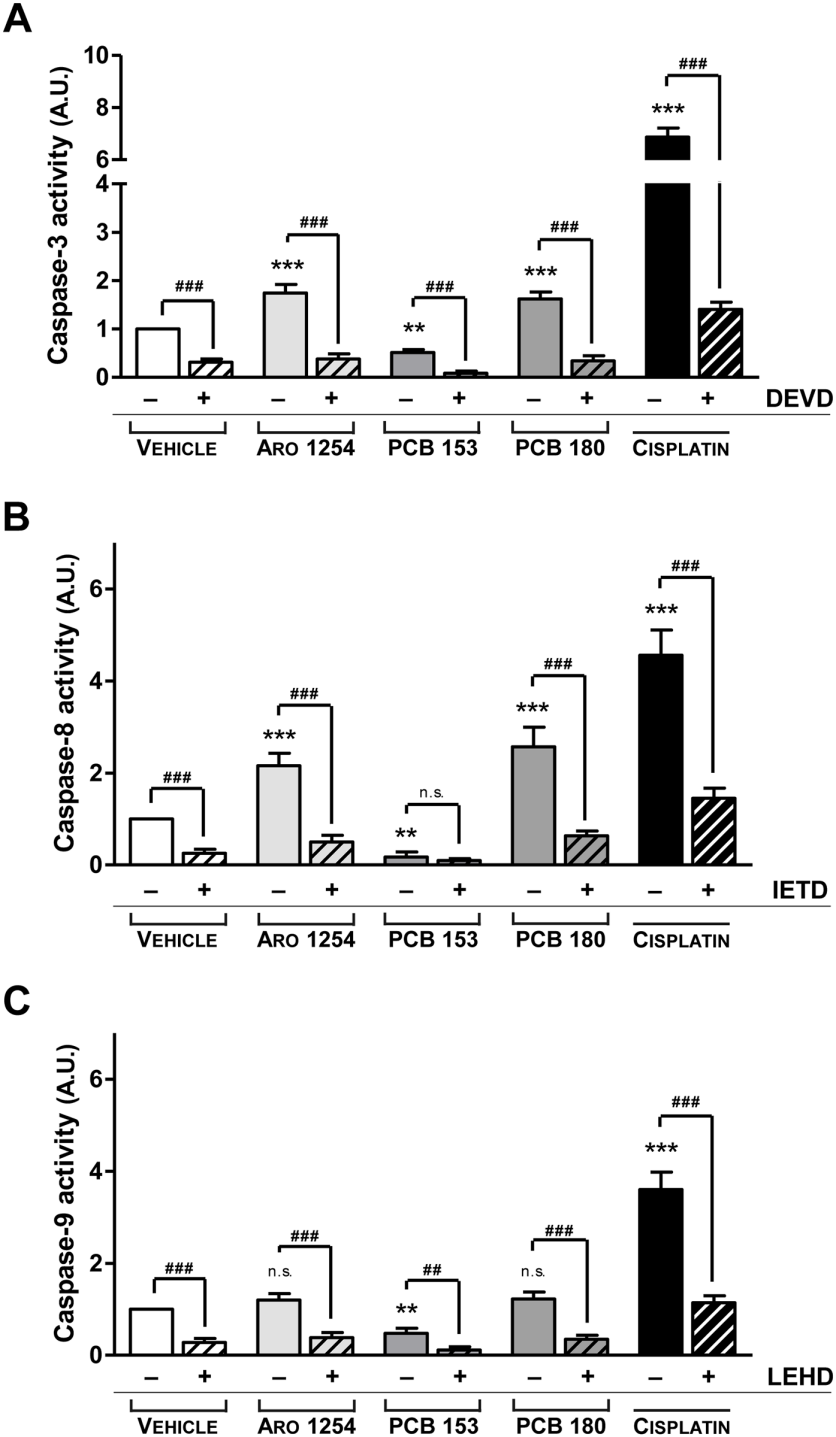
Changes in the activity of caspases during exposure to PCBs. Caspase activity was assessed by fluorimetric or luminometric assays, as described in the Materials and Methods. Cisplatin (32 μM) was included as a positive control. The activity of the three individual caspases decreased by exposure to PCB 153 (A, B, C). On the other hand, the activity of both caspase-3 and caspase-8 increased after treatment with Aroclor 1254 or PCB 180 (A, B), while the activity of caspase-9 was unaffected (C). To confirm the specificity of PCB action on caspases, cells were also pre-treated with caspase inhibitors prior to PCB exposure. 20 μM Ac-DEVD-CHO (A), Ac-IETD-CHO (B) or Ac-LEHD-CHO (C) were used as inhibitors of caspase -3, -8 or -9, respectively. Data (mean ± SD of five independent experiments) were expressed as arbitrary units (A.U.); the value of 1 was attributed to the caspase activity of vehicle-exposed cells. **, p<0.001, ***, p<0.0001, compared with the vehicle, according to ANOVA followed by Dunnett’s post hoc test. ##, p<0.005, ###, p<0.0005, p = n.s. (not significant), according to Student’s t-test adjusted for multiple comparisons following the Bonferroni method.

**Fig 6:**
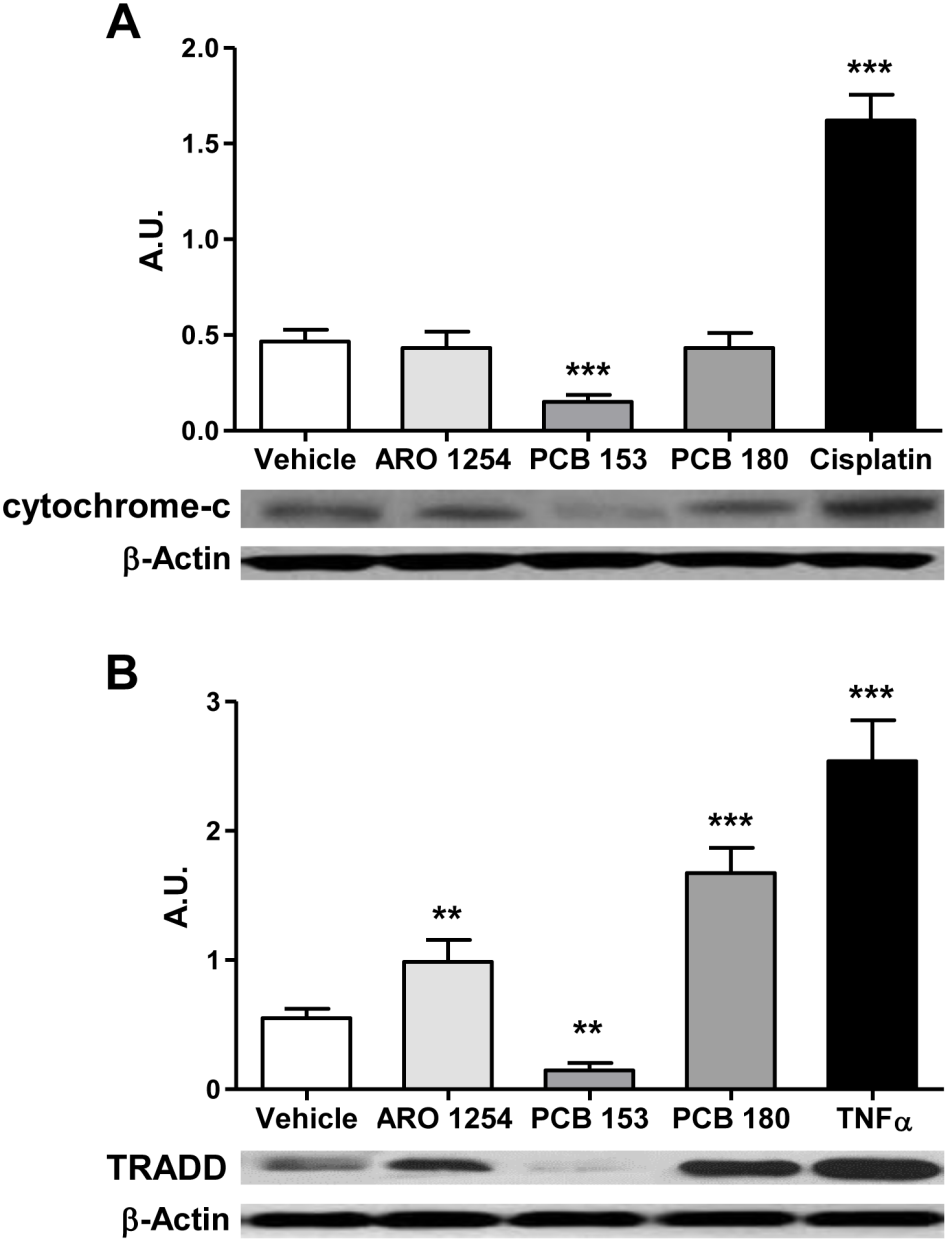
Effects of PCBs exposure on the molecular mechanism of apoptosis. Representative Western blot from pituitary primary cell extracts obtained as described in the Materials and Methods. Primary pituitary cells were incubated for 24 hours with fresh serum-free medium containing the test substances at a 10 μM concentration or the vehicle (DMSO 0.1%). The expression of both the cytochrome c, as regulator of caspase-9 (intrinsic mechanism), and TRADD, as regulator of caspase-8 (extrinsic mechanism) were evaluated (A, B). Cisplatin (32 μM) or TNFα (10 μM) were used as positive controls for cytochrome c or TRADD, respectively. In line with the results on caspases expression and activity, both mechanisms were hindered by PCB 153 treatment. TRADD expression also increased by cell exposure to Aroclor 1254 or PCB 180, while cytochrome c expression remained unaltered. Data are expressed as arbitrary units (A.U.), which represent the ratio between the intensity of the band of interest and the intensity of the band corresponding to the control protein (β-actin). The mean ± SD of measurements obtained in five independent experiments are reported. **, p<0.001, ***, p<0.0001 compared with vehicle, according to ANOVA followed by Dunnett’s post hoc test.

To explore the molecular mechanism of apoptosis, primary pituitary cells were exposed only to those PCBs that affected this process (Aroclor 1254, PCB 153 or PCB 180). Both expression and activity of caspase-8 and -9 (involved in the extrinsic and in the intrinsic apoptosis pathways, respectively) and of caspase-3 were evaluated (Figs 4 and 5). The expression of caspase-3, changed according to the changes in apoptosis observed after the exposure of cells to PCBs, showing a significant increase after treatment with Aroclor 1254 or PCB 180, and a decrease after the exposure to PCB 153 (Fig 4A). Aroclor 1254 and PCB 180 increased the expression of caspase-8 (Fig 4B; p<0.0001), whereas the expression of caspase-9 was unchanged (Fig 4C). PCB 153, however, significantly reduced the expression of both caspase-8 and caspase-9 (Fig 4B and 4C). More importantly, the activity of the three caspases changed according to their expression levels, when exposed to Aroclor 1254 or individual non-dioxin-like congeners (Fig 5A–5C). In contrast, no effect was observed in the presence of selective caspases inhibitors, (Fig 5A–5C), indicating the specificity of caspases regulation by non-dioxin-like PCBs. In addition, the expression of cytochrome c, as regulator of caspase-9, was hindered by PCB 153 treatment and remained unchanged after exposure to Aroclor 1254 or PCB 180 (Fig 6A). The expression of TRADD on the other hand, as a regulator of caspase-8, was also hindered by PCB 153 and was increased by Aroclor 1254 or PCB 180 (Fig 6B), in line with the results on caspases expression and activity. As positive controls to induction of cytochrome c or TRADD expression, cisplatin (32 μM) and Tumor Necrosis Factor (TNF) α (1 μM) were used, respectively [48, 49] (Fig 6A and 6B).

**Fig 7:**
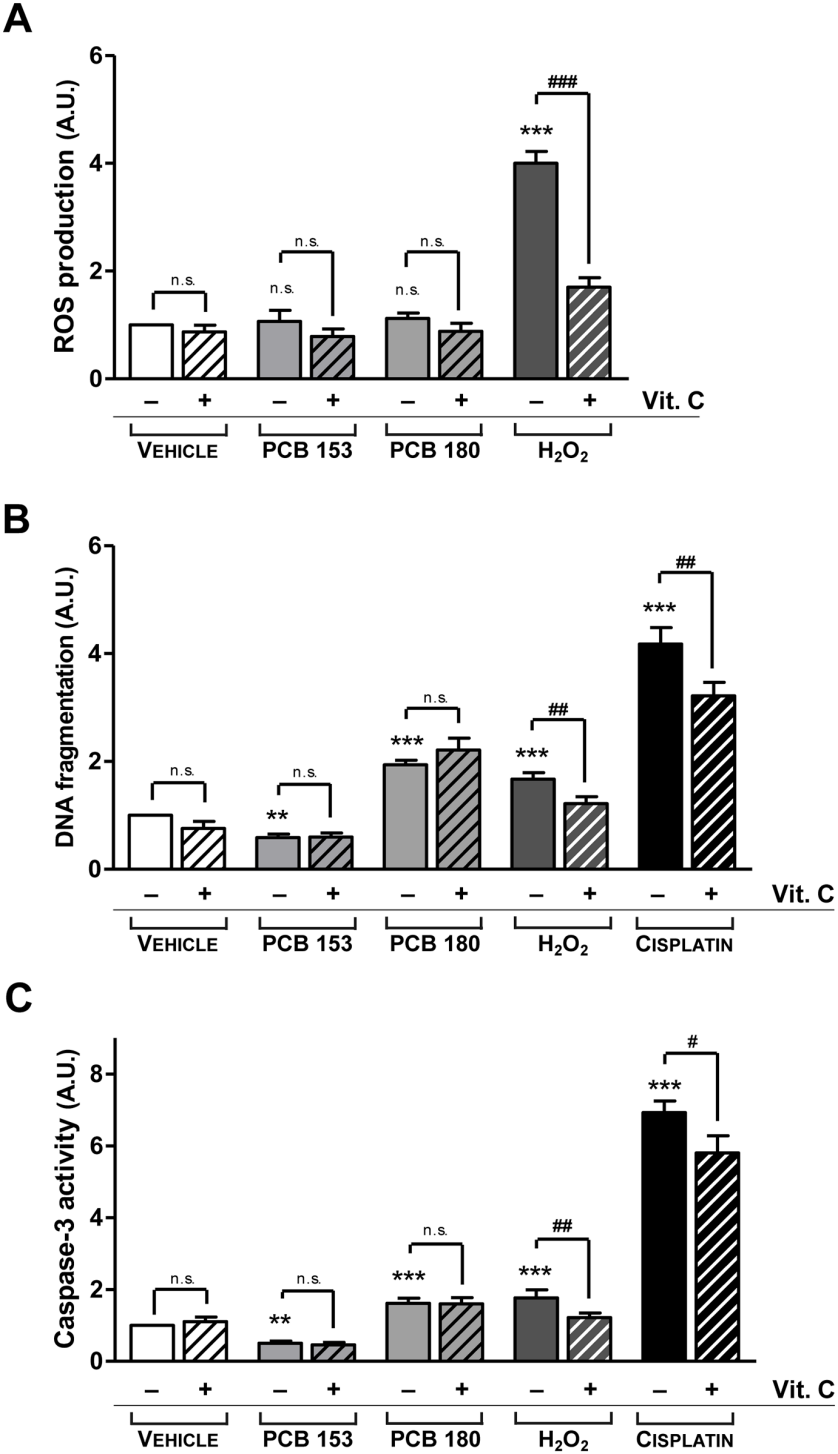
Evaluation of apoptosis induced by ROS production. Quantitative ROS generation was measured using the oxidant-sensitive probe dichlorofluorescein (H2DCF-DA) assay (A), apoptosis was evaluated by ELISA DNA fragmentation assay (B), and caspase-3 activity was determined by fluorimetric assay (C), as described in the Materials and Methods. Pituitary primary cells were treated as described in the study design. 10 μM Hydrogen Peroxide (H_2_O_2_) (A), 50 μM H_2_O_2_ (B, C), or 32 μM Cisplatin (B,C) were used as positive controls. In all the assays, 1 mM Ascorbic Acid (vitamin C) was used as an antioxidant. ROS production was not influenced by non-dioxin-like PCB 180 or PCB 153, with or without the addition of vitamin C (A). Moreover, vitamin C had no effect on either apoptosis or caspase-3 activity of PCBs-treated pituitary cells (B, C), suggesting that ROS do not play a key role in PCBs-mediated apoptosis mechanism. Results represent the mean ± SD of five independent experiments, each performed in quadruplicate and expressed as arbitrary units (A.U.). The value of 1 was assigned to ROS production (A), DNA fragmentation (B) or caspase-3 activity (C) from vehicle-exposed cells. **, p<0.001, ***, p<0.0001 compared with vehicle, according to ANOVA followed by Dunnett’s post hoc test. #, p<0.05, ##, p<0.005, ###, p<0.0005, p = n.s. (not significant) according to Student’s t-test adjusted for multiple comparisons following the Bonferroni method.

Since PCBs have been reported to increase intracellular ROS content [50, 51], we evaluated whether PCBs-mediated changes of ROS intervene in the regulation of pituitary cells apoptosis. As shown in Fig 7A, the ROS levels did not change when cells were exposed to individual PCBs, either alone or in combination with vitamin C, a ROS production inhibitor. Likewise, ROS production was not associated either with changes in pituitary cell DNA fragmentation level or with changes in caspase-3 activity (Fig 7B and 7C). These data suggest that both Aroclor 1254 and PCB 180 induced the apoptosis of pituitary cells acting through the extrinsic pathway. On the other hand, the congener PCB 153 reduced apoptosis affecting both the extrinsic and the intrinsic pathways. Interestingly, ROS did not seem to be involved in the PCBs-mediated apoptosis of pituitary cells.

### The effect of the inhibition of Aryl-hydrocarbon receptor, CYP1A1, or thyroid hormone receptor on apoptosis

**Fig 8:**
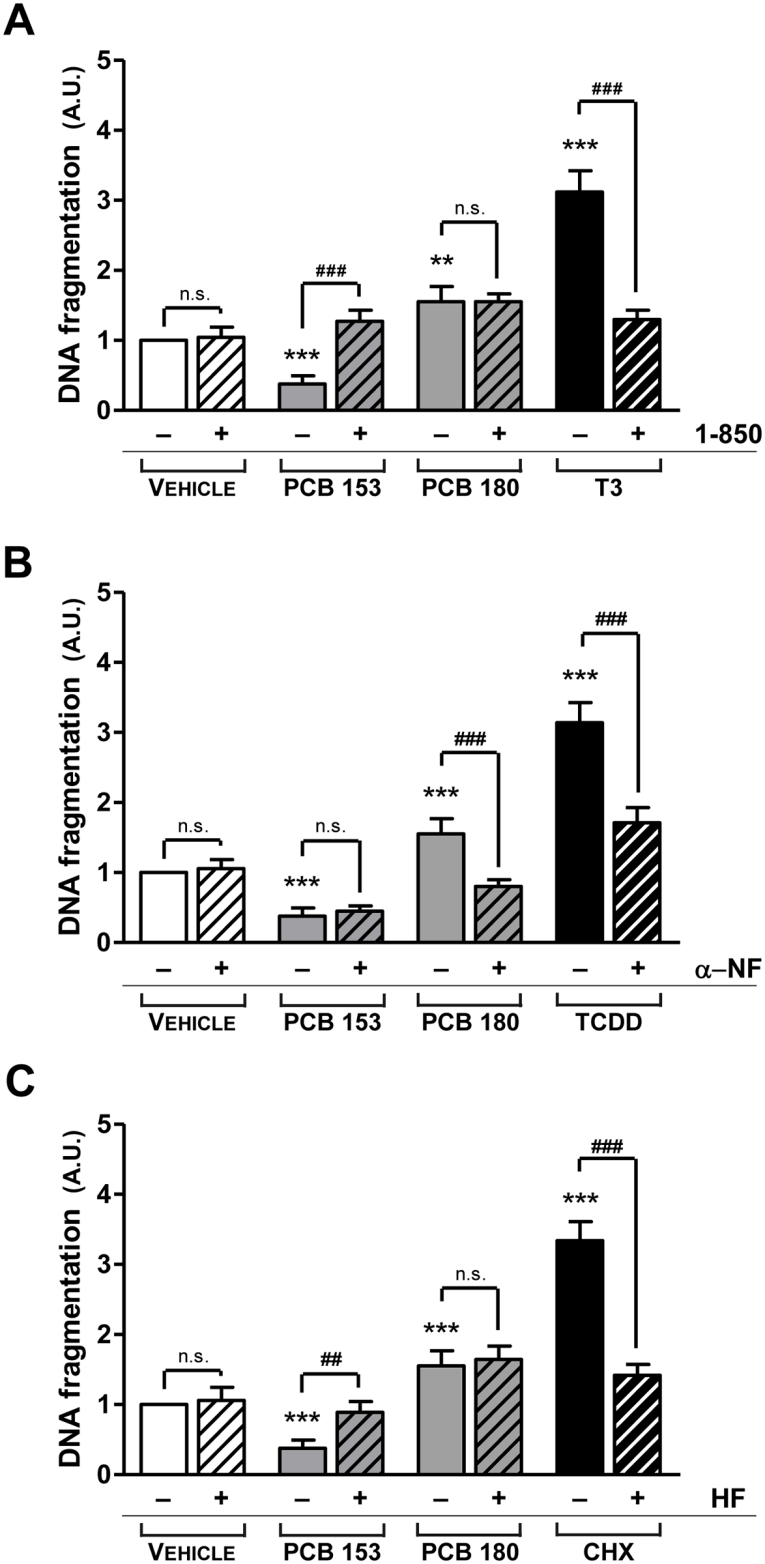
The pathways involved in the regulation of apoptosis by PCBs. Apoptosis was evaluated in the absence or presence of a thyroid hormone receptor antagonist, 1–850, or an AhR inhibitor, α-Naphthoflavone (α-NF) or a CYP1A1 inhibitor, 7-Hydroxyflavone (HF), using the ELISA DNA fragmentation test, as reported in the Materials and Methods. Only individual congeners that modified apoptosis (PCB 153 and PCB 180) were tested. As a positive control to the induction of TR-, AhR- or CYP1A1-mediated apoptosis, T3 (10 nM) (A), TCDD (3 μM) (B) and CHX (10 μM) (C) were used. Primary pituitary cells were pretreated with 1–850, α-NF or HF antagonists (20 μM) for 30 min before adding the vehicle or PCB 153 or PCB 180 (10 μM). Blocking TR or CYP1A1 action with a selective inhibitor, abolished the anti-apoptotic effect of PCB 153 (A and C). In contrast, an AhR antagonist abolished the pro-apoptotic effect of PCB 180 (B). Data represent the mean ± SD of five independent experiments, each performed in quadruplicate and expressed as arbitrary units (A.U.), assuming that the DNA fragmentation level obtained from the cells exposed to the vehicle was considered as 1.**, p<0.001, ***, p<0.0001 compared with vehicle, according to ANOVA followed by Dunnett’s post hoc test. ##, p<0.005, ###, p<0.0005, p = n.s. (not significant), according to Student’s t-test adjusted for multiple comparisons following the Bonferroni method.

To explore whether PCBs-induced changes in apoptosis involve TR, AhR or CYP1A1, cells were exposed to congeners that affected apoptosis (PCB 153 or PCB 180), after the addition of a specific inhibitor of TR (1–850), AhR (α-NF) or CYP1A1 (HF), respectively, to the culture medium. As a positive control to the induction of TR-, AhR- or CYP1A1-mediated apoptosis, T3 (10 nM), TCDD (3 μM) and CHX (10 μM) were used, as previously described [52–54]. The anti-apoptotic phenotype of pituitary cells due to PCB 153 was overridden by the use of both TR and CYP1A1 inhibitors (Fig 8A and 8C), but not by the AhR inhibitor (Fig 8B). The pro-apoptotic effect of PCB 180 was not changed by the addition of TR or the CYP1A1 inhibitor (Fig 8A and 8C), whereas it was abolished by the AhR inhibitor (Fig 8B).

These data suggest that non-dioxin-like PCBs may intervene in the regulation of apoptosis of pituitary cells by interaction with both the TR and AhR functions, leading to an anti-apoptotic or a pro-apoptotic effect, respectively.

### The effect of PCBs on pituitary cells proliferation

**Fig 9:**
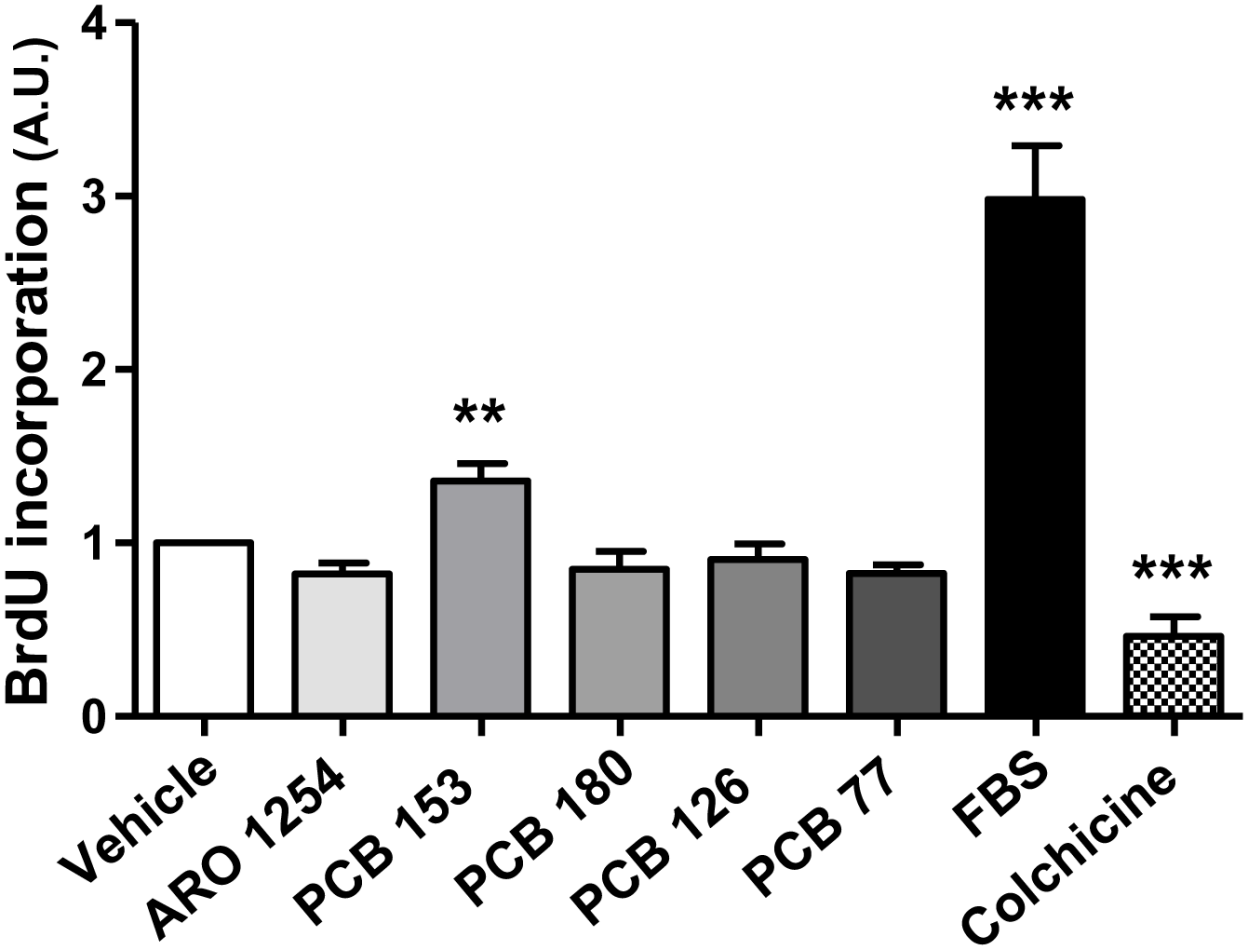
The 5-bromo-2’-deoxyuridine uptake. Cell proliferation was evaluated by measuring the cellular uptake of 5-bromo-2’-deoxy-uridine, in the presence of individual congeners (PCB 153, PCB 180, PCB 126, PCB 77) or the Aroclor 1254 mixture (ARO), by BrdU incorporation ELISA colorimetric assay, as detailed in the Materials and Methods. Primary pituitary cells were incubated for 24 hours with fresh serum free-medium containing the test substances at 10 μM concentration or with vehicle (DMSO 0.1%). The culture medium, supplemented with 10% fetal bovine serum and Colchicine (1 μM) were used as a positive or negative control, respectively. Only in the presence of the non-dioxin-like congener PCB 153, did the uptake of 5-bromo-2’-deoxy-uridine increase, suggesting the potential growth of pituitary cells exposed to this congener. Data represent the mean ± SD of five independent experiments, each performed in quadruplicate and expressed as arbitrary units (A.U.), assuming that the BrdU incorporation level obtained from the cells exposed to vehicle was considered as 1. **, p<0.001, ***, p<0.0001 compared with vehicle, according to ANOVA followed by Dunnett’s post hoc test.

Aroclor 1254 or the non-dioxin-like congener PCB 180 left the incorporation of 5’-bromo-2’-deoxyuridine by exposed cells unchanged. A similar result was obtained using the dioxin-like congeners PCB 77 and PCB 126. In contrast, the incorporation of 5’-bromo-2’-deoxyuridine increased by 30% after exposure to PCB 153, compared to the control (Fig 9; p<0.001). The culture medium, supplemented with 10% fetal bovine serum, used as positive control, caused a highly significant induction of cells proliferation (p<0.0001), whereas colchicine, used as a negative control, blocked the cells proliferation, as expected.

These data suggest that the reduced apoptosis caused by PCB 153 may have the potential to increase the risk of pituitary cells proliferation.

## Discussion

PCBs are persistent pollutants, which may disrupt the endocrine function through multiple mechanisms [55]. From a structural point of view, PCBs with a minimum of four chlorines in the lateral positions and none or only one in the ortho positions, have similar effects to those of dioxin. The other congeners are referred to as non-coplanar or ortho-substituted, and are considered as non-dioxin like [56]. A structure-function relationship of PCBs has been proposed [57].

Dioxin-like congeners exert their effects by binding to the AhR, similarly to dioxin [8]. In contrast, non-dioxin-like PCBs act through different mechanisms that do not directly involve the AhR pathway.

AhR is a cytoplasmic receptor which, after binding to a ligand, translocates to the nucleus, dimerizes with the AhR nuclear translocator and binds to xenobiotic response elements, thus modulating the transcription of sensitive genes [58].

The action of non-dioxin-like congeners is less clearly defined and may involve multiple mechanisms. In fact, it has been reported that non-dioxin like PCBs can affect the binding of the thyroid hormone to transthyretin, act as androgen receptor antagonists, or inhibit gap junctional intercellular communication [59]. These actions may contribute to the onset of hypothyroidism or hypogonadism, or may promote neoplasms [2, 4].

The effects of PCBs on apoptosis have been evaluated in several tissues, including the liver, neural cells, ovary, testis, breast, and spleen [11, 16, 18, 60–62]. Overall, studies have reported that PCB 153 consistently reduced apoptosis [20–22]. In contrast, Aroclor 1254 or Aroclor 1221 increased apoptosis in the spleen or hypothalamus [10, 62]. PCB 132 increased apoptosis in testis. PCB 28, PCB 10, and PCB 187 reduced apoptosis only when it was induced by ultraviolet rays in rat liver primary culture; whereas they had no effect on spontaneous apoptosis [11, 16].

Our data show the effects of non-dioxin-like PCBs on the spontaneous apoptosis of the primary culture of pituitary cells, suggesting that pituitary cells may be a target for selected non-dioxin-like PCBs, whereas they are not affected by dioxin-like congeners. In fact, the Aroclor 1254 mixture induced apoptosis, similarly to the non-dioxin-like PCB 180, both activating caspases 3 and 8. Surprisingly, the dioxin-like congeners PCB 77 and PCB 126, which have been reported to increase apoptosis in the liver [63] and in HL-60 splenocyte cell culture [62], were silent in the pituitary cells. This suggests a tissue specific action of non-dioxin-like congeners in the modulation of apoptosis.

**Fig 10:**
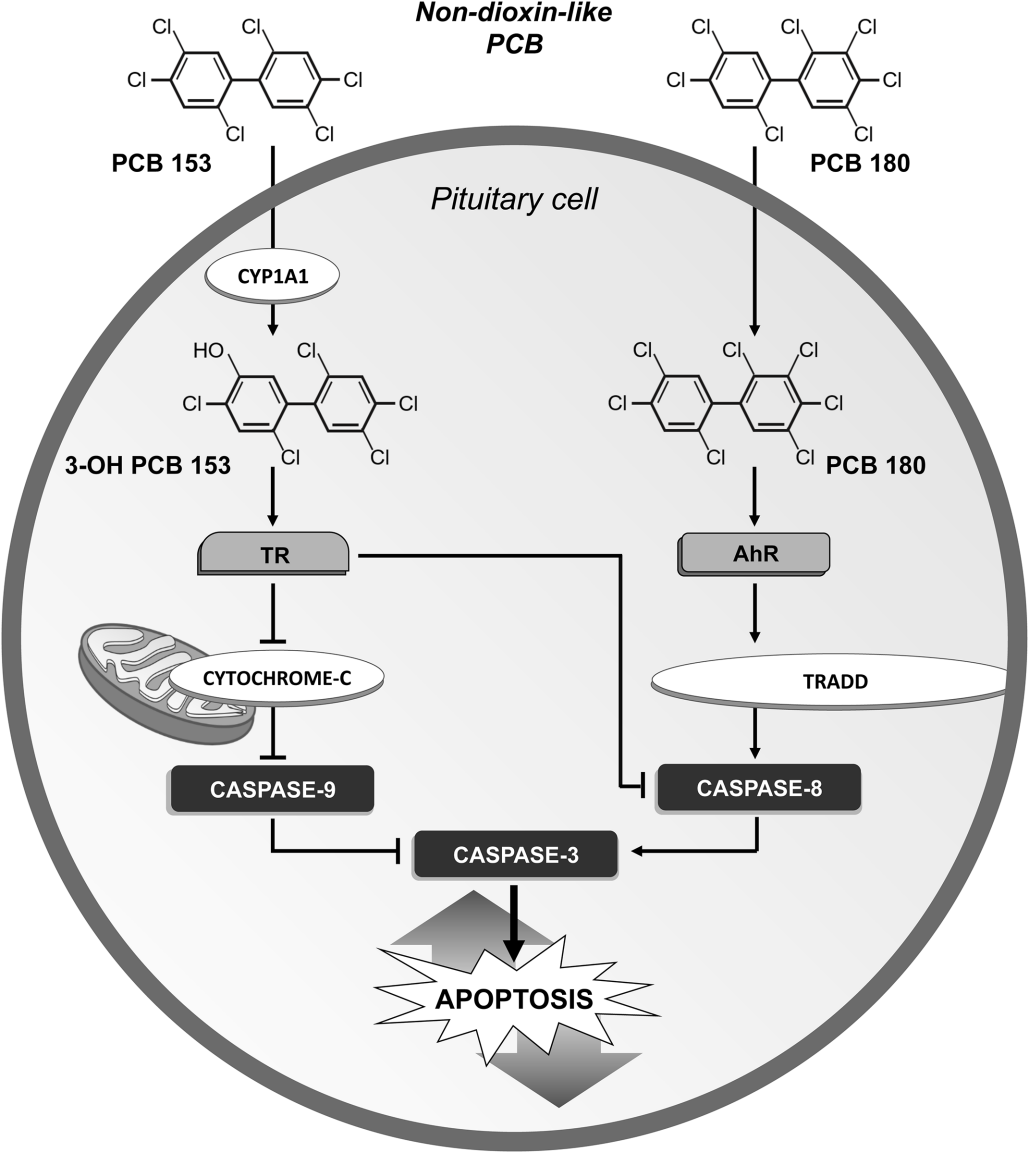
Proposed mechanism regulating the apoptotic pathway by PCBs in pituitary cells. The non-dioxin-like PCB 180 increased apoptosis by activating the extrinsic pathway (TRADD, caspase-8). In contrast, the extrinsic (TRADD, caspase-8) and intrinsic (cytochrome c, caspase-9) pathways were down-regulated by the non-dioxin-like PCB 153, leading to an anti-apoptotic and putative proliferative phenotype. Changes in apoptosis by PCBs occur through the interference of PCB with TR, AhR and CYP1A1 action. A proposed mechanism is shown featuring the disruption of TR action by PCB 153 after its hydroxilation by CYP1A1, leading to an anti-apoptotic phenotype. In contrast, the disruption of AhR by PCB 180 led to a pro-apoptotic phenotype.

The role of non-dioxin-like congeners in the regulation of apoptosis in the pituitary cells was further confirmed by the inhibition of apoptosis and the reduction of expression and activity of caspases 3, 8 and 9 by PCB 153. Thus, the non-dioxin-like congener PCB 180 may induce apoptosis, mainly by regulating the extrinsic pathway, whereas the anti-apoptotic phenotype induced by PCB 153 could be obtained by blocking both the intrinsic and the extrinsic pathways. This view was further supported by the increased expression of TRADD by PCB 180 and the reduced expression of both cytochrome c and TRADD by PCB 153 (Fig 10).

Recent data suggest that ROS may have a direct role in mediating death receptor activation and the mithocondrial pathway after exposure to Aroclor 1254 [51, 64]. In addition, chondrocytes exposed to non-dioxin-like PCBs have been reported to undergo several changes, including necrosis and apoptosis, mediated, in part by oxidative stress [65]. It has also been reported that AhR ligand 5F203 induces ROS-mediated DNA damage and apoptosis in human breast cancer cells [66]. However, our data showed that ROS production was not affected in pituitary cells exposed to PCBs, suggesting that cell- specific mechanisms play a significant role in PCB-induced apoptosis.

Multiple pathways seem to be involved in the regulation of apoptosis induced by PCBs in the pituitary gland. It has been reported that the thyroid hormone (TH) may up-regulate the expression of 4-1BB that encodes a TNF receptor superfamily protein which, in turn, activates caspases to induce apoptosis [67]. However, the cellular response to TH is dependent on cell type. In fact, in Xenopus laevis, TH induced apoptosis in larva-proper cells and proliferation in adult progenitor cells [68] In our cellular model, the thyroid hormone receptor seems to be involved in the reduction of apoptosis caused by non-dioxin-like PCBs, as shown by data obtained with the TR antagonist, which abolished the apoptosis inhibition induced by PCB 153. The block of CYP1A1 was also associated with the abrogation of the anti-apoptotic phenotype induced by PCB 153. Thus, to disrupt TR action on apoptosis, PCB 153 should first be hydroxilated by CYP1A1, as previously suggested [69, 70]. However, the independent and direct effect of CYP1A1 on pituitary cells apoptosis cannot be ruled out. In fact, the reduction in CYP1A1 expression may play a critical role in the inhibition of apoptosis and proliferation [71].

Although AhR is usually activated by dioxin-like PCBs, we found that AhR may be involved in the regulation of pituitary apoptosis induced by non-dioxin-like PCB 180. Furthermore, PCB 180 shares several toxicological targets with dioxin-like compounds [72]. Thus, it is not surprising that PCB 180 may affect the AhR pathway in the pituitary apoptosis. In fact, AhR agonists may sensitize the keratinocytes to apoptosis. In contrast, AhR knock-out mouse hepatocytes or fibroblasts are resistant to apoptosis [73, 74]. Mechanisms underlying apoptotic changes by AhR activation may involve the expression of AhR sensitive genes. In fact, a constitutively active AhR mutant in human leukemic cells [75] increases the expression of caspase-8 and apoptosis-related genes, through a xenobiotic response element (XRE)-dependent mechanism.

The reduction in apoptosis is considered as an early event of tumorigenesis [12]. The pro-tumorigenic effects of PCBs have been studied mainly in rat liver, and evidence suggests a promoting effect in cells previously initiated with a genotoxic carcinogen [2, 76]. PCBs are thus generally considered as tumor-promoting agents, which may drive the clonal expansion of altered or pre-neoplastic cells. A similar mechanism has been proposed for rat hepatocytes [16], where non-dioxin-like PCBs suppressed only UV-induced apoptosis but had no effect on the spontaneous apoptosis. In our model, the non-dioxin-like PCB 153 reduced spontaneous apoptosis, suggesting that pituitary cells may be even more sensitive to PCBs. In addition, the significant increase in 5-bromo-2’-deoxyuridine incorporation during PCB 153 exposure, suggests that normal pituitary cells exposed to this congener have the potential to grow.

## Conclusions

In conclusion, the pituitary gland seems to be a target of non-dioxin-like PCBs, which promote either a pro-apoptotic or an anti-apoptotic phenotype, depending on their chemical structure. Overall, our data show that the regulation of pituitary apoptosis by PCBs involves multiple pathways, occurring through an AhR or a TR-dependent mechanism, and is associated with changes in the expression level and activity of caspases.

In addition, our results may indicate that, in the pituitary gland, the anti-apoptotic effects of the non-dioxin-like PCB 153 could be associated with its capacity to modulate cell proliferation, thus highlighting the potential role of this pollutant in tumor-promoting activities.
